# Potential Protective Effect of Carotid Endarterectomy: Inducing Ischemic Tolerance in Brain Tissue after Stroke

**DOI:** 10.1007/s12031-025-02470-0

**Published:** 2026-02-03

**Authors:** Rastislav Mucha, Marek Furman, Alexandra Urbanova, Ivan Kopolovets, Miroslava Nemethova, Michal Virag, Stanislav Hresko, Vladimir Katuch, Vladimir Sihotsky

**Affiliations:** 1 Institute of Neurobiology of Biomedical Research Center, Slovak Academy of Sciences, Soltesovej 4, 040 01 Kosice, Slovakia; 2 Department of Vascular Surgery, Eastern Slovak Institute of Cardiovascular Diseases and Faculty of Medicine, Pavol Jozef Safarik University, Kosice, Ondavska 8, 040 01 Kosice, Slovakia; 3 Department of Animal Nutrition and Husbandry, University of Veterinary Medicine and Pharmacy in Kosice, Komenskeho 78, 041 81 Košice, Slovakia; 4 Department of Neurosurgery, Faculty of Medicine, Trieda SNP 1, 040 11 Košice, Slovakia; 5 Present address: Department of Cardiac and Vascular Surgery, Kardiocentrum Agel, Kosice-Saca, Lucna 57, 04015 Košice, Slovakia

**Keywords:** Brain stroke, Ischemic tolerance, Gene expression, Blood, Human

## Abstract

Stroke is a serious disease, ranking among the leading causes of mortality and permanent disability in EU countries. The ischemic cascade, triggered by the blockage of oxygenated blood supply to brain tissue, leads to excitotoxicity, oxidative stress, inflammation, and eventually, cell death. Current research highlights the promising neuroprotective effects of conditioning, which induces ischemic tolerance (IT). Thus, the main objective of this study is to analyze selected genes affected by ischemic stroke and the neuroprotective response to ischemic stroke, with a focus on ischemia and ischemic tolerance in peripheral blood. We investigated changes in gene expression indicative of cerebral ischemia during carotid endarterectomy (CEA), a procedure that involves the temporary occlusion of the *arteria carotis interna*. To assess the influence of CEA on IT induction, we performed a whole-transcriptome analysis of peripheral blood cells isolated from symptomatic (791 DEGs in correlation with negative control), asymptomatic (688 DEGs in correlation with negative control), and oximetric (637 DEGs in correlation with negative control) patients. The presence of gene expression changes in genes selectively identified through whole-transcriptome analysis was subsequently statistically verified. Using quantitative qRT-PCR, we monitored gene expression changes in10 genes *SLC2A14, TRPM7, UGP2, PLLP, ND4L, HMSD, SESN3, DPY19L4, UBE3A,* and *PCDH9*. The results suggest that CEA affected the expression of all monitored genes, with statistically significant differences between groups, indicating the activation of distinct ischemic tolerance cascades in different patient groups. These findings may contribute to a better understanding and characterizing of the molecular mechanisms underlying ischemic tolerance.

## Highlights

Whole transcriptome expression changes were identified in stroke

A potential method of neuroprotection activation after stroke was identified

Potential blood-based markers of stroke and neuroprotection were identified

## Introduction

Stroke is now the second most prevalent cause of mortality in the European Union, following ischemic heart disease, with its incidence increasing by 26.1% since 1995. Together, stroke and ischemic heart disease have caused 15.2 million deaths per year (15–15.6 million) (Doeppner et al. 2018). Moreover, 47.4 million disability-adjusted life years (DALYs) were recorded in 2013 (Shi et al. 2018). In addition, an increase in incidence is expected (Doeppner et al. 2017). Over 50% of stroke survivors require assistance from others to carry out their daily tasks. Stroke places a significant financial burden on caregivers and healthcare systems. In Europe, stroke-related expenses exceed 38 billion euros per year (Howard et al. 2021).

The mechanisms of ischemia are complex and form the so-called ischemic cascade. The ischemic cascade can be broadly described as cellular bioenergetic failure resulting from focal hypoperfusion of the brain’s neural tissue, followed by excitotoxicity, oxidative stress, blood-brain barrier dysfunction, microvascular damage, haemostatic activation, post-ischemic inflammation, and ultimately, cell death of neurons, glial cells, and endothelial cells (Brouns and De Deyn 2009). Given this complexity, therapies targeting individual ischemic mechanisms have not been effective so far. Therefore, attention has shifted to endogenous neuroprotective mechanisms and their ability to induce ischemic tolerance. This process, known as conditioning, begins with stimulation that triggers the production of effector molecules, which either alter gene expression or modify existing proteins. These changes result in an ischemic-tolerant phenotype. Ischemic tolerance (IT) refers to a state in which cells exhibit resistance to the harmful effects of ischemia, leading to a reduced rate of cell death caused by ischemia-reperfusion (IR) injury. Cells exposed to metabolic stress or sublethal ischemia become temporarily resistant to a subsequent otherwise lethal level of stress (Burda et al. 2023). Ischemic conditioning, as an effective non-pharmacological strategy for reducing IR injury, was first demonstrated in 1986 in the field of cardiology on a dog’s heart (Murry et al. 1986). The neuroprotective mechanism of reperfusion injury in conditioning involves the suppression of pathophysiological pathways that occur during ischemia. As a result, conditioning offers an innovative approach to neuroprotection by targeting multiple cellular and molecular processes, including apoptosis, inflammation, oxidative stress, brain oedema, hemodynamics, and neurorepair (Saccaro et al. 2021; Furman et al. 2023).

Carotid endarterectomy (CEA) is a surgical procedure used to remove atherosclerotic plaque. Performing this procedure requires the temporary occlusion of the carotid artery. Subsequently, during CEA, the brain is supplied with blood only through one of the carotid arteries. In some cases, CEA can lead to a decrease in brain tissue oxygenation above 20%, which may symptomatically manifest in patients as a mild ischemic attack. These symptoms spontaneously resolve within 2 to 7 days. Such a stimulus activates a cellular response (a cascade of reactions inducing ischemic tolerance), which is reflected in changes at the gene expression level and the subsequent de novo synthesis of effector proteins.

In experimental animal models of ischemic stroke, it has been shown that a certain gene expression profile after ischemia occurs in both peripheral blood and brain nerve tissue (Tang et al. 2001). Peripheral blood appears to be a good choice as a source of RNA (Furman et al. 2022). Clear similarities in gene expression in both blood and brain were found in a study of ischemic stroke performed by focal occlusion of the middle cerebral artery (MCA), in a model of monkey *Macaca mulatta*. The brain structures of this species are significantly similar to those of the human brain. In this analysis, it was found that 493 upregulated and as many as 2156 downregulated genes overlap, with this overlap in gene expression in both brain nerve tissue and blood reached a statistically significant level for both upregulated and downregulated gene groups (Ramsay et al. 2019).

The aim of this work was to identify the effect of CEA on the process of IT induction based on transcriptomic analysis of peripheral blood cells and subsequently study the changes in the expression of selected genes in the transcriptome of peripheral blood cells of patients influenced by CEA as a potential neuroprotective post-conditioning in humans.

## Material and Methods

### Composition of the Test Cohorts

The set of test cohorts chosen for our research analysis consisted of a total of 22 volunteers, of whom 20 were patients and 2 were healthy individuals. Each participant was properly informed about the goal and course of the study. They voluntarily agreed to participate in this biomedical study, as confirmed by signing the informed consent form. In addition, each participant voluntarily provided data on their medical history for the purposes of the study. Preoperative (before CEA) and postoperative (2 days after CEA) neurological examinations were performed for each participant. CRP levels typically increase transiently immediately after surgery, preoperative CRP levels and levels measured 48 hours postoperatively are more predictive of early restenosis, underscoring the key role of inflammation in adverse outcomes. Based on this evidence, patients with CRP levels ≥2 mg/L were excluded from the study. After carotid endarterectomy, IL-6 levels generally rise as a result of surgery-induced inflammation. However, there is no universally defined “normal range,” as values vary across individuals and studies. Baseline IL-6 levels in healthy individuals are typically low (approximately 1–3 pg/mL), whereas post-CEA levels may transiently increase. Previous studies have reported baseline values around 1.23 pg/mL with higher postoperative or local levels, and elevated IL-6 has been associated with more severe plaque characteristics and an increased risk of complications, such as new lesion formation. Cutoff values around 2.0 pg/mL are commonly used for risk stratification; therefore, in this study, a threshold of 2.0 pg/mL was applied for sample exclusion. To assess the severity of stenosis, imaging techniques such as ultrasonography (USG), MRI angiography, digital subtraction angiography (DSA), and CT angiography were used. The degree of internal carotid artery stenosis for each patient was evaluated using at least two imaging techniques. The size of the ischemic lesion for each patient who had suffered an ischemic stroke prior to CEA was measured either with MRI or a native CT brain scan. CEA was recommended for all patients in accordance with current clinical guidelines. The study participants were divided into four groups:

Asymptomatic Group (Asym) – a cohort of 8 asymptomatic patients, meaning patients recommended for CEA based on current clinical guidelines, without symptomatic manifestations of stroke.

Symptomatic Group (Sym) – a cohort of 8 symptomatic patients, meaning patients recommended for CEA based on current clinical guidelines for those who underwent CEA between 7 and 180 days after the onset of neurological symptoms of ischemic stroke.

Oximetric Group (Oxim) – 4 patients (both Sym and Asym) in whom a decrease in saturation detected by transcranial Doppler of more than 20% was observed during CEA.

Negative Control (Negat) – the control group consisted of 2 healthy volunteers (ages 27 and 49) without a history of acute or chronic diseases and therefore without the need for medication.

The average age of the participating patients was 71 years, and efforts were made to ensure that the distribution of age and gender was similar across all patient groups. The duration of carotid artery occlusion during the surgical procedure, recorded by the Invos device, was also documented in the anesthesiology surgical record.

For patients included in the tested groups the following exclusion criteria were applied: TIA, presence of perioperative inflammatory processes, history of oncological or any embolic disease, autoimmune diseases, history of thrombosis or other types of ischemic events excluding stroke, acute and chronic kidney or liver diseases, acute or chronic infections, coagulation disorders, ischemic lesions (if larger than 3 cm). As an exclusion factor for the asymptomatic group, we also included stroke that occurred more than 6 months ago.

Patient categorization based on symptomatology was performed in accordance with the guidelines of the European Society for Vascular and Endovascular Surgery regarding the treatment of atherosclerotic disease of the carotid and vertebral arteries. These guidelines are routinely applied in clinical practice to assess the severity of carotid stenosis. The severity of stenosis was determined using the NASCET method, which calculates the degree of carotid narrowing based on the residual luminal diameter at the stenosis, measured by the observer, relative to the diameter of a disease-free segment of the carotid artery proximal to the stenosis, with approximately parallel vessel walls.

A statistical power analysis was performed to evaluate the sensitivity of the study design. Given the total number of participants (*n* = 22), including 20 patients undergoing carotid endarterectomy (CEA) and 2 healthy volunteers, the effective sample size for paired pre- and postoperative comparisons was *n* = 20. Using a two-tailed paired t-test with a significance level of α = 0.05, the statistical power (1 – β) was estimated according to Cohen’s method and verified using the G*Power 3.1 software (Faul et al. 2009).

Negative control group was included primarily as a technical and biological reference rather than as a group intended for formal statistical comparison with patient cohorts. Due to the small sample size of the control group, meaningful statistical adjustment for age and sex (e.g., multivariable regression or stratification) was not feasible and would not be statistically valid.

Within the patient cohorts, efforts were made to ensure comparable distributions of age and sex across the asymptomatic, symptomatic, and oximetric groups, with an overall mean age of 71 years and no intentional sex imbalance. Consequently, age- and sex-related confounding is expected to be minimal for comparisons among patient subgroups, although we recognize that the limited sample size restricts definitive conclusions.

For this sample size, the detectable standardized effect size (Cohen’s d) corresponding to a power of 0.80 was approximately d = 0.63, indicating that the study was adequately powered to detect medium-to-large effects, but powered for detecting small differences in preliminary study. Subgroup analyses (Asymptomatic, Symptomatic, Oximetric, and Control groups) had markedly lower power due to small group sizes (Table 1). Consequently, these subgroup comparisons should be interpreted as exploratory and hypothesis-generating rather than confirmatory (Serdar et al. 2021).

**Table 1:** Clinical characteristic of the patients

Patient groups	Symptomatic	Asymptomatic	Oximetric	Total
Number of patients	8	8	4	20
Age (years)	75	68	71	71
Sex (male/female)	50%	40%	50%	45%
Hypertension	8	8	4	20
Heart disease	5	5	2	12
Renal insufficiency	0	0	0	0
Pulmonary diseases	1	0	0	1
Diabetes mellitus	2	2	1	5
Smokers	2	2	1	5
Clamping time (minutes)	14	15	16	15
Oximetry decline (%)	14	15	26	18

Overview of patients included in the study cohorts and clinical characteristics for each of them.

All patients received exactly the same postoperative care according to the same protocol, which included antibiotic treatment, low molecular weight heparin (0.05 ml per 10 kg body weight every 12 hours), and an antiplatelet agent (75 mg of clopidogrel once daily). During this postoperative care, patients also continued taking their chronic medications. No comorbidities (including TIA, presence of perioperative inflammatory processes) or medications were recorded that could have affected the gene analysis results or that were not evenly represented across the patient groups. Each patient included in our study underwent the reverse type of surgical procedure known as carotid endarterectomy (CEA). All patients underwent standardized postoperative monitoring following carotid endarterectomy, including neurological examination performed 2 days after surgery, as well as routine clinical and laboratory surveillance as part of standard perioperative care. Minor postoperative changes consistent with expected surgical recovery (e.g., transient local inflammation at the surgical site) were not considered as complications and did not require additional intervention. Importantly, all patients included in the final analysis met the predefined exclusion criteria regarding inflammatory, neurological, and systemic conditions, thereby minimizing confounding effects on the measured molecular and clinical parameters.

The implementation of CEA as well as the blood sample collection took place at the Department of Anesthesiology and Intensive Care of the Eastern Slovak Institute of Cardiovascular Diseases, Inc., Kosice, Slovakia (VUSCH, Inc.) and also at the Department of Vascular Surgery of VUSCH, Inc., Blood samples of a total volume of 5 ml for the purposes of biomedical research were collected from patients with the assistance of nurses, alongside routine blood draws and preoperative examinations. The Ethics Committee of VUSCH, Inc., after reviewing the purpose and methodology of the study, decided on July 30, 2018, to grant approval for the proposed project.

### Microarray Analysis

Whole-transcriptome microarray analysis of representative samples of isolated, purified, and decontaminated human mRNA (RIN scores of 7–10 were accepted for further RNA analysis) was performed using Human Clariom-S plates (Affymetrix) designed for human samples. Post-processing analysis of the measured data was conducted using Power Tools software (Affymetrix).

The data were summarized and normalized using the SST-RMA (Signal Space Transformation-Robust Multichip Analysis) method implemented in Affymetrix® Power Tools (APT). The results were exported with SST-RMA analysis at the gene level, and a differential gene expression (DEG) analysis was performed.

The statistical significance of expression level data was determined using “fold change.” For the set of differentially expressed genes (DEG), hierarchical cluster analysis was performed using complete linkage and Euclidean distance as a measure of similarity.

Gene enrichment and functional annotation analyses for generating a list of significant probes were conducted using Gene Ontology (http://geneontology.org) and KEGG (http://kegg.jp).

All data analyses and visualizations of differentially expressed genes were performed using R 3.3.2 (www.r-project.org).

Samples were analyzed using the Affymetrix Clariom™ S Assay. Raw intensity data (CEL files) were pre-processed and normalized using the Signal Space Transformation-Robust Multichip Analysis (SST-RMA) algorithm implemented in Affymetrix Power Tools (APT). Normalization included background correction, probe summarization, and log2 transformation. Data quality was assessed using box plots, density plots, hierarchical clustering, and multidimensional scaling (MDS) plots, and Pearson’s correlation coefficients were calculated to confirm sample reproducibility. All samples showed high correlation, indicating consistent data quality.

Potential batch effects were evaluated through hierarchical clustering, MDS, and correlation analysis. No significant batch effects were observed, and therefore no batch correction was applied.

Differentially expressed genes (DEGs) were identified based on fold change (|FC| ≥ 2) across comparisons between test and control samples. Functional enrichment analysis was performed using g:Profiler, and statistical significance of enriched Gene Ontology terms was assessed using False Discovery Rate (FDR) correction according to the Benjamini–Hochberg method, with terms considered significant at adjusted *p* *value* <0.05.

This approach ensured robust identification of DEGs and minimized the risk of false positives due to multiple testing.

### Isolation and Processing of Blood Samples, mRNA Isolation, Reverse Transcription to cDNA, and Primer Design

Peripheral whole blood was collected into Eppendorf tubes containing an anticoagulant solution, and plasma and blood cells were then separated by centrifugation for 15 minutes at 3500 g at 4 °C. The individual fractions were stored at −80 °C.

Phosphate buffer (PBS), composed of 137 mmol/L NaCl, 10 mmol/L Na₂HPO₄·2H₂O + KH₂PO₄, and 2.7 mmol/L KCl, with a pH of 7.4, was used to dilute whole blood cells in a 1:1 ratio. The diluted sample was lysed using TRIzol reagent (Thermo Fisher, USA), and total RNA was extracted with chloroform and isopropanol (propan-2-ol). To eliminate any potential genomic DNA contamination of mRNA, the lysate was treated with DNase I (RNase-free enzyme, Thermo Fisher Scientific, USA). The High-Capacity cDNA Reverse Transcription Kit (Applied Biosystems, USA) was used to reverse transcribe RNA to cDNA according to the manufacturer’s instructions.

Primer design was performed in silico using the Geneious software (Biomatters, Ltd., New Zealand). The designed forward and reverse primer sequences are listed in Table 2.

**Table 2:** Primers for qRT-PCR quantification used in the study

Gene	Forward primer [bp]	Revers primer [bp]	Annealing temperature [°C]	Amplified sequence [bp]
*18S*	GACCATAAACGATGCCGACT	GTGAGGTTTCCCGTGTTGAG	60	190
*ADM*	TTGGACTTTGCGGGTTTTGC	TTTCGGAACTGCGAGGAAGT	60	200
*CDKN1A*	TTGTCGCTGTCTTGCACTCT	CCTGACCCACAGCAGAAGAA	60	200
*GADD45G*	ATGAAGATGACGACCGGGAC	TAGCGACTTTCCCGGCAAAA	60	271
*IL6*	CAGGAACGAAAGTCAACTCCA	ATCAGTCCCAAGAAGGCAACT	60	94
*TM4SF1*	TGTGCTATGGGAAGTGTGCA	TTTATTTGTTTTTGTTTTTT	60	819
*SLC2A14*	ACCCCAGCTCTGATCTTTGC	GCCACAGACAAGGACCAGAG	58.6	185
*UGP2*	GGCAAACTGAGACTGGTGGA	ATGACATTCAGGCCTCCATCC	58.6	200
*PLLP*	GTCTTCCCTCCCTGCATTTCA	CCCATGTGCCTAGTCAGCAA	69	180
*DPY19L4*	TAGTCCCAGCTACTCCGAGG	GTCTGGCATTTGGGAGGCTA	60	190
*TRPM7*	TGTCTTGGTGGGGCATGGTG	CTGACCTTGTGATCCGCCCA	67	220
*ND4L*	CGCTCACACCTCATATCCTCCC	CGTAGTCTAGGCCATATGTGTTGGA	60	202
*SESN3*	TTGACACAACCATGCTGCGC	TGAGTGTTTGAACTGCCGCCA	62.3	200
*HMSD*	ATCAACCCCCTTGCAGCCAG	TAGCAAACCCGGCAACCTCTG	60	215
*UBE3A*	TGTCACCGAATGGCCACAGC	CGTGCAGGCTTCATTTCCACAG	61.5	
*PCDH9*	CGCTGTCGCCATGCATCAAG	CCGGCAGGCTTATTGTCCCA	67	214

Forward and reverse primer sequences along with qPCR reaction parameters: annealing temperature (primer binding) and the length of the amplified sequence during the qRT-PCR reaction in base pairs [bp].

### Quantitative Real-Time PCR (qRT-PCR)

The cDNA concentrations were diluted to the level recommended by the manufacturer of the qRT-PCR mastermix used (Power SYBRTM Green PCR Master Mix, Applied Biosystems, USA).

The recorded Ct values of individual genes were normalized according to the Ct values of the housekeeping gene used as the reference for the given sample. Subsequently, the ΔCt values for specific genes were calculated. The housekeeping gene used was 18S. The PCR reaction was performed on the Bio-Rad CFX96™ Real-Time PCR Detection System, with the software used for evaluating Ct values being CFX Maestro qPCR Analysis Software (Bio-Rad).

The normalized ΔCt value for a specific gene in the sample was obtained by calculation:

ΔCt(gene x) =/Ct(gene x)/–/Ct(*18S*)/.

Ct – Cycle threshold.

ΔCt - The normalized value of a specific gene x in the sample.

*18S* - Housekeeping gene.

The resulting values of the overall expression change of the studied gene relative to the negative samples of the control groups (ΔΔCt(gene x)) were calculated as the difference in ΔCt(gene x) values between the tested groups (test) vs. their negative samples of the control group (ctrl).

ΔΔCt(gene x) = ΔCt(gene x test) – ΔCt(gene x ctrl).

ΔCt - The normalized value of a specific gene x in the sample.

ΔΔCt - The final value of the overall expression change.

The gene expression level (fold difference (fd)) was calculated based on the following equation:

fd = 2^-ΔΔCt^.

ΔΔCt - The final value of the overall expression change.

The final values of RNA expression change were obtained based on calculation:

RNA (expression change rate) = log2 (fd).

fd – fold change.

Primer efficiency was evaluated prior to analysis, and only primers demonstrating efficiencies greater than 90%, corresponding to near-exponential amplification with approximate doubling of product per cycle, were included in the study. Amplification specificity was confirmed by melt curve analysis conducted at 65 °C for 5 seconds followed by 95 °C for 5 seconds. All quantitative PCR reactions were performed in triplicate. Outlier data points were excluded based on predefined criteria, including the presence of technical errors, statistical deviation, and aberrant amplification or melting curve profiles.

### Statistical Evaluation

For calculating the variance of ΔCt(gene x) values in the set of individuals, we used the mathematical formula for standard deviation (s):

$$s=\sqrt{\frac{1}{N-1}\ast {\sum}_{i=1}^N{\left( xi-\overline{x}\right)}^2}$$

s - standard deviation.

N - Number of individuals in the tested group.

𝑥𝑖 - The ΔCt(gene x) value for a specific sample.

$\overline{x}$ - The average value of ΔCt(gene x) from the entire tested group.

The standard deviation was calculated as a 95% confidence interval. For the calculation, we used the online software Standard Deviation Calculator (calculator.net).

Using the t-test, we calculated the size of the difference between the sample means in relation to their variability, with a threshold value and statistical significance (P) based on the chosen significance level (0.05 to 0.001):

*P* > 0,05 - Insignificant, P 0,05–0,01 (*) - Marginally significant, P 0,01–0,001 (**) - Moderately significant, *P* < 0,001 (***) - Highly significant.

## Theory/Calculation

We hypothesize that carotid endarterectomy (CEA) induces specific changes in gene expression within the transcriptome of peripheral blood cells, which are associated with the mechanisms of induced tolerance (IT) and may indicate the activation of post-ischemic neuroprotection in patients undergoing CEA.

Intervention in cerebral perfusion through CEA may trigger biological responses similar to ischemic preconditioning. It is further hypothesized that these responses are reflected at the gene expression level in peripheral blood, allowing for non-invasive monitoring of the activation of potentially protective mechanisms. Transcriptomic analysis may reveal gene expression signatures related to neuroprotection, inflammatory response, oxidative stress, or apoptosis—key pathways commonly implicated in induced tolerance and postconditioning.

## Results

### Whole Transcriptome Analysis

**Fig. 1:**
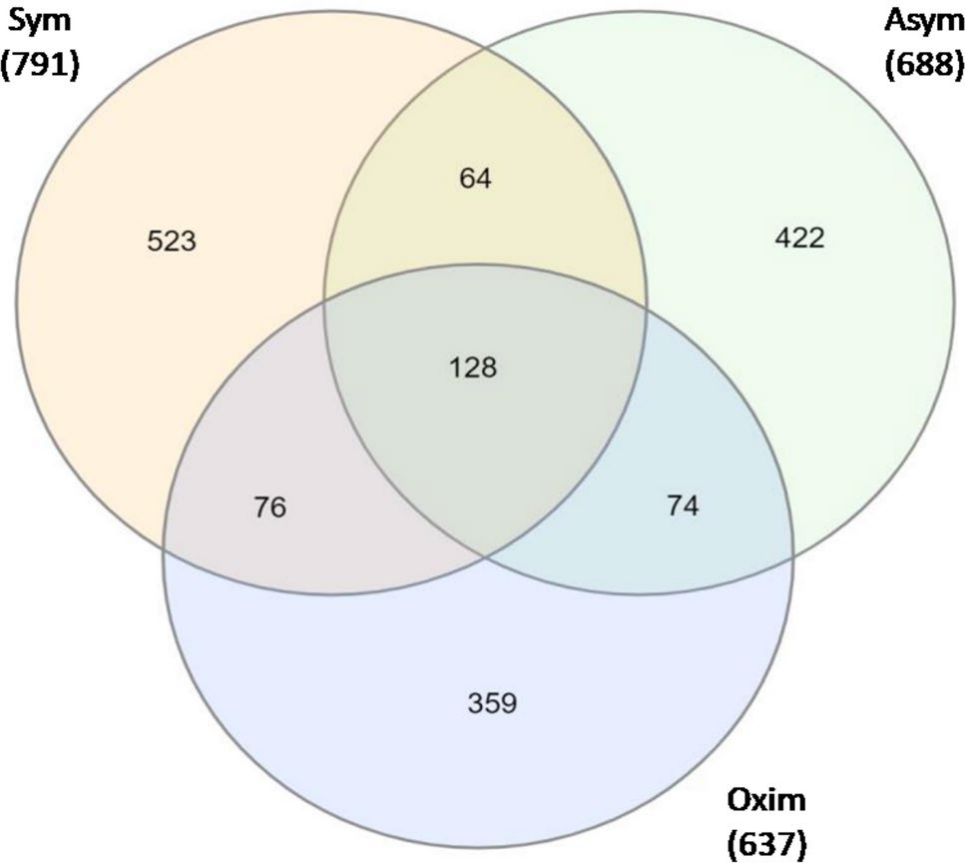
Microarray analysis results – Venn diagram. All numbers showed in the figures means Differentially expressed genes (DEGs)

Using microarray whole transcriptome analysis, we identified the occurrence of 791 genes with a significant change in expression levels > ±2 compared to the negative control within the Sym group, with 523 genes being specific to this group and not overlapping with other groups. The total number of genes with a significant change in expression levels > ±2 compared to the negative control in the Asym group was identified as 688 genes, with 422 genes being specific to this group and not overlapping with others. For the Oxim group, the total number of genes with a significant change in expression levels > ±2 compared to the negative control was 637, with 359 genes being specific to this group and not overlapping with others (Fig. 1).

Graphical representation of the number of genes and their overlap within patient groups 48 hours after carotid endarterectomy, measured by microarray analysis of representative samples from these groups. The total number of genes with a significant change in expression levels > ±2 compared to the negative control is shown for each group in parentheses under the group name. The individual gene counts for the overlap between groups are expressed numerically directly on the graph.

### Gene Analysis

This part of the study focused on analyzing changes in gene expression levels selected based on the results of the previous whole-transcriptome microarray analysis of representative samples from individual patient test groups undergoing CEA, who had experienced a stroke at varying time intervals before CEA. Based on the expression levels of individual genes within the groups, we identified the genes with the highest expression changes compared to the negative control for each group. In our selection process, we also considered the involvement of these genes in molecular signaling pathways related to ischemic brain tissue damage or, conversely, to tolerance mechanisms against such damage. The genes selected according to the above-mentioned criteria are listed in Table 3.

**Table 3:** Genes selected for a detailed analysis of expression level changes

Patient groups	Upregulated gene	Downregulated gene
Symptomatic		*SLC2A14*
(Sym)	*UGP2*	*TRPM7*
		*UBE3A*
		*PCDH9*
Asymptomatic	*PLLP*	*UBE3A*
(Asym)	*ND4L*	*PCDH9*
Oximetric	*HMSD*	*UBE3A*
(Oxim)	*SESN3*	*PCDH9*
	*DPY19L4*	

Based on the results of the whole transcriptome analysis of gene expression changes in representative patient samples, we selected the genes with the greatest difference in expression levels compared to the negative control for each group. Upregulated genes exhibited a significantly increased expression level, whereas downregulated genes showed a significant decrease in expression.

Sym – symptomatic patients;

Asym – asymptomatic patients;

Oxim – patients with a decrease in oximetry of more than 20% during CEA.

We primarily focused on observing changes caused by a decrease in oximetry of more than 20% during CEA. The results were normalized against the negative control and compared with Sym and Asym patients also after CEA.

#### Expression of Genes Selectively Analyzed in the Symptomatic Group

**Fig. 2:**
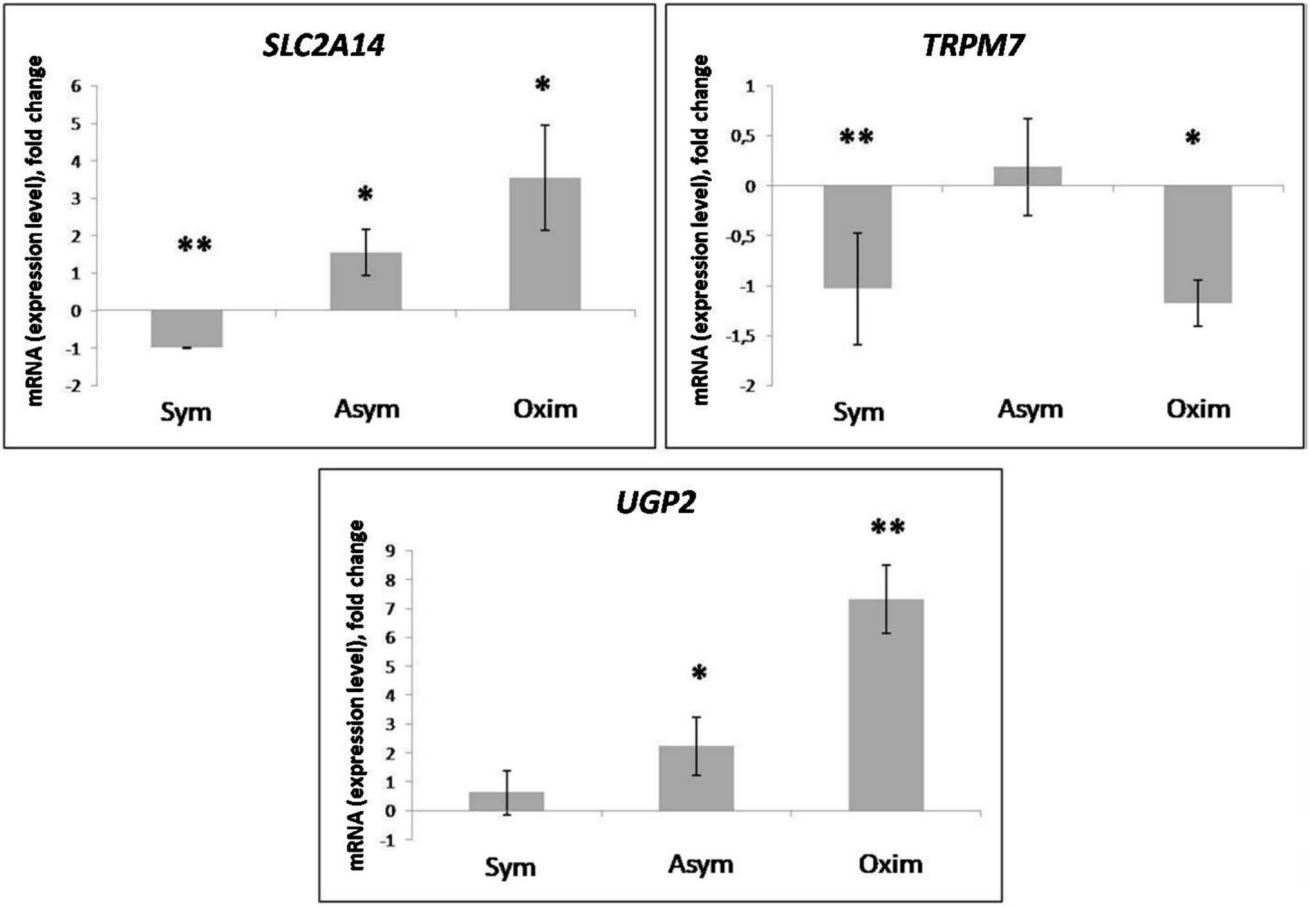
Expression levels of *SLC2A14, TRPM7*, and *UGP2* modified by the impact of CEA. Comparison of expression level changes in *SLC2A14, TRPM7*, and *UGP2* among the Sym, Asym, and Oxim groups that underwent CEA. All changes were normalized against the negative control, which represents the 0 value on the X-axis in the graphs. For statistically significant expression changes: * *P* < 0.05, ** *P* < 0.01, *** *P* < 0.001

The increased expression level of *SLC2A14* compared to the negative control was observed in the Asym and Oxim groups in a marginally significant form, whereas in the Sym group, we observed a statistically significant decrease in *SLC2A14* expression compared to the negative control (Figs. 2 and 6).

An increase in *TRPM7* expression compared to the negative control was observed only in the Asym group, though it was not significant. However, a significant decrease in *TRPM7* expression compared to the negative control was observed in both the Sym and Oxim groups, with a higher level of statistical significance in the Sym group (Fig. 2).

The expression level of *UGP2* increased compared to the negative control in all tested groups. The least pronounced change was observed in the Sym group. A statistically more significant increase in *UGP2* expression was observed in the Asym group. The most substantial increase in expression was recorded in the Oxim group, where the statistical significance reached *P* < 0.01 (Figs. 2 and 6).

#### Expression of Genes Selectively Analyzed in the Asymptomatic Group

**Fig. 3:**
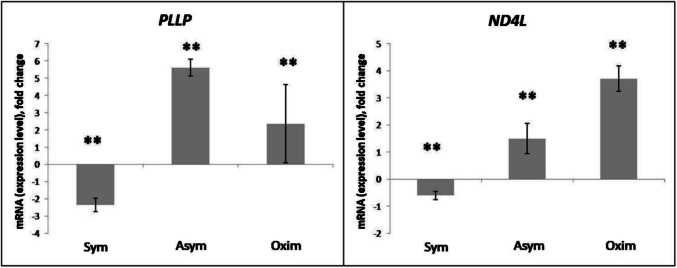
Expression levels of *PLLP* and *ND4L* genes modified by the impact of CEA. Comparison of expression level changes in *PLLP* and *ND4L* genes among the Sym, Asym, and Oxim groups that underwent carotid endarterectomy (CEA). All changes were normalized against the negative control, which represents the 0 value on the X-axis in the graphs. For statistically significant expression changes: * *P* < 0.05, ** *P* < 0.01, *** *P* < 0.001

The most pronounced increase in *PLLP* expression compared to the negative control was observed in the Asym group, with a milder increase also observed in the Oxim group. In both groups, the results were statistically significant in comparison to the negative control (P < 0.01). In contrast, in the Sym group, the *PLLP* expression was significantly decreased when compared to the negative control (*P* < 0.01) (Figs. 3 and 6).

An increase in *ND4L* expression compared to the negative control was observed in the Asym and Oxim groups, with a more pronounced increase recorded in the Oxim group. A slight decrease in *ND4L* expression compared to the negative control was observed in the Sym group. The statistical significance of the results was set at *P* < 0.01 for all groups (Figs. 3 and 6).

#### Expression of Genes Selectively Analyzed in the Oximetric Group

**Fig. 4:**
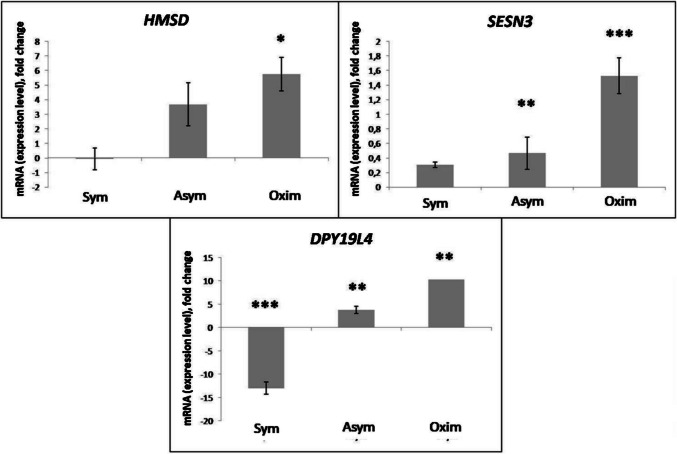
Expression levels of *HMSD, SESN3*, and *DPY19L4* genes modified by the impact of CEA. Comparison of expression level changes in *HMSD*, *SESN3*, and *DPY19L4* genes among the Sym, Asym, and Oxim groups that underwent carotid endarterectomy. All changes were normalized against the negative control, which represents the 0 value on the X-axis in the graphs. For statistically significant expression changes: * *P* < 0.05, ** *P* < 0.01, *** *P* < 0.001

An increase in *HMSD* expression compared to the negative control was observed in both the Asym and Oxim groups, with a more pronounced and statistically significant increase in the Oxim group (*P* < 0.05). In the Sym group, the measured *HMSD* expression level was similar to the negative control and, therefore, not statistically significant (Figs. 4 and 6).

An increased expression level of *SESN3* compared to the negative control was observed in all groups, with the most statistically significant increase observed in the Oxim group (*P* < 0.001), a less pronounced increase in the Asym group (*P* < 0.01), and without statistically significant change in *SESN3* expression observed in the Sym group (Figs. 4 and 6).

A significant increase in *DPY19L4* expression compared to the negative control occurred in a milder form in the Asym group (*P* < 0.01) and in a more pronounced form in the Oxim group (*P* < 0.01). A decrease in *DPY19L4* expression compared to the negative control was observed in the Sym group (*P* < 0.001) (Figs. 4 and 6).

#### Selectively Analyzed Downregulated Genes Common to all Groups

**Fig. 5:**
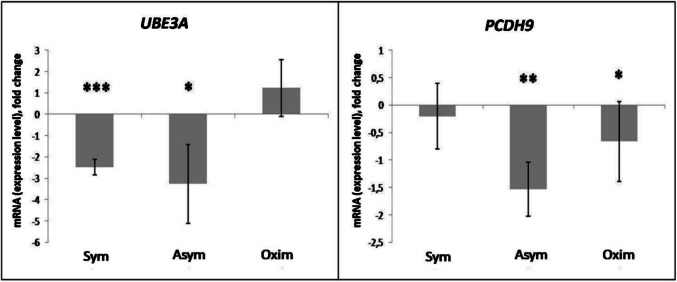
Expression levels of *UBE3A* and *PCDH9* genes modified by the impact of CEA. Comparison of expression level changes in *UBE3A* and *PCDH9* genes among the Sym, Asym, and Oxim groups that underwent carotid endarterectomy. All changes were normalized against the negative control, which represents the 0 value on the X-axis in the graphs. For statistically significant expression changes: * *P* < 0.05, ** *P* < 0.01, *** *P* < 0.001

**Fig. 6:**
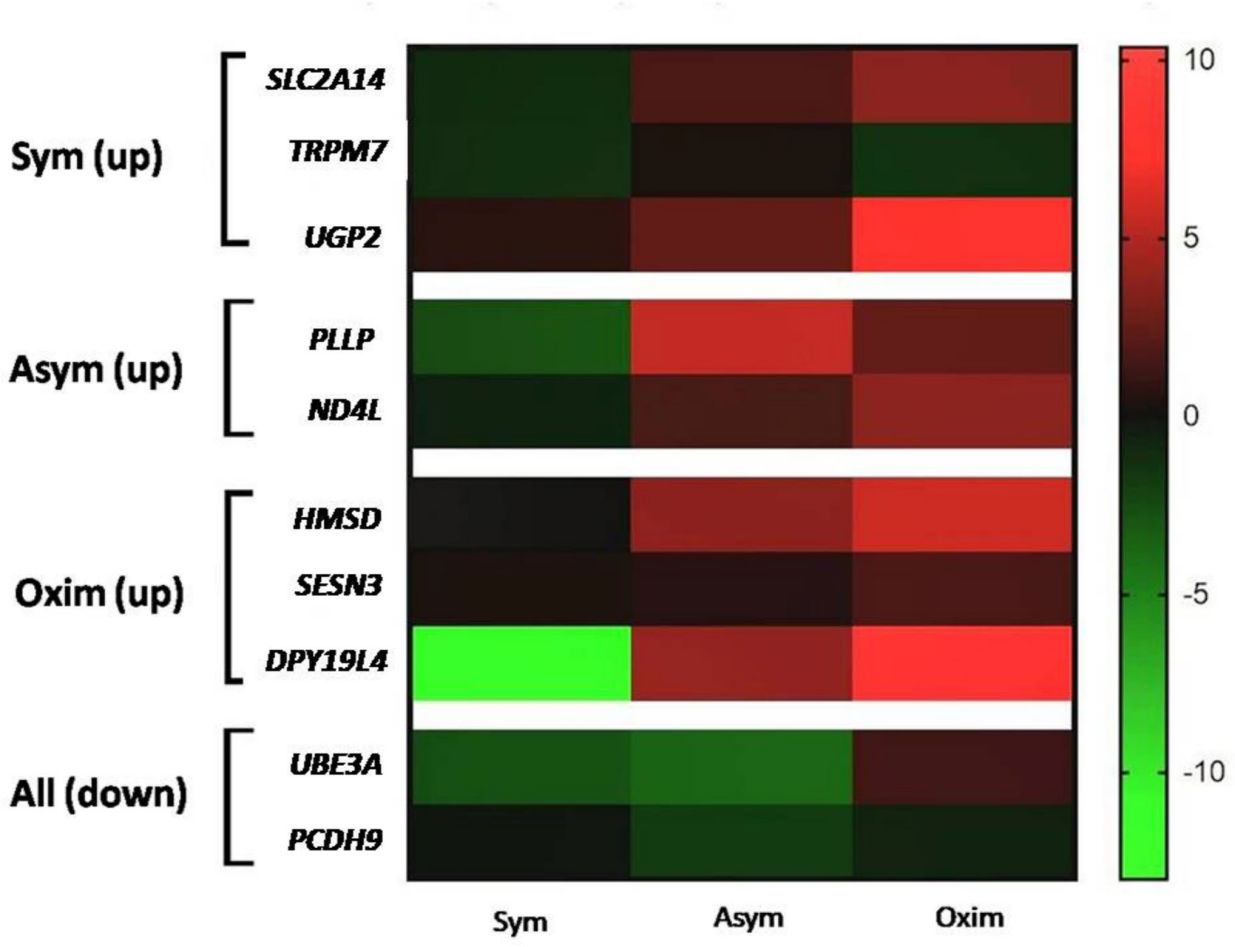
Gene expression heatmap based on gene analysis

A decrease in *UBE3A* expression compared to the negative control was observed in the Sym and Asym groups, with the result in the Sym group being statistically more significant. In the Oxim group, a slight, statistically insignificant increase in *UBE3A* expression compared to the negative control was observed (Figs. 5 and 6).

A decrease in *PCDH9* expression compared to the negative control was observed in all tested groups. The most pronounced decrease in expression was recorded in the Asym group, a less pronounced decrease in the Oxim group, and the least pronounced decrease in the Sym group. The level of statistical significance decreased in the same order, from the most significant result in the Asym group with a statistical significance of *P* < 0.01 to the Sym group, which showed no statistical significance (Figs. 5 and 6).

The heatmap graphically represents the gene expression ratio (left column) relative to the three studied patient groups: Sym, Asym, and Oxim. The colors in the heatmap (right column) indicate the level of gene expression compared to the control sample. Green represents decreased expression, while red indicates increased expression compared to the control sample. The control sample, with a value of 0, is marked in black. The color gradients between green and red represent varying degrees of expression changes. The arrangement of genes within the table corresponds to the results of the representative samples analyzed using the microarray method, summarized in Table 3. The genes of each representative sample are separated by white horizontal sections, with their descriptions listed on the far left. The expected upregulation (up) or downregulation (down) of each gene within the group is indicated in parentheses next to the group name.

## Discussion

For the whole transcriptome microarray analysis on representative samples from each tested cohort, our study used the Human Clariom-S chip from Affymetrix, which contains over 20,000 probes of well-annotated genes that bind to specific mRNA sequences. This allows for the simultaneous analysis of a large number of genes and their expression in one experiment. From the Venn diagram of the microarray analysis results, we can see that when compared to the negative control, hundreds of genes were identified as specifically expressed in our tested cohorts out of the more than 20,000 genes. When comparing overlaps between the individual groups, only tens of genes were involved. Since the microarray analysis only involved representative samples, a rational selection of the most suitable gene candidates was performed for subsequent, more detailed qRT-PCR analysis within broader statistical patient groups.

The specific selectively chosen genes and their quantitative identification of expression changes were performed by group, based on the results of the microarray analysis. The genes induced in the microarray analysis results specific to the Symptomatic group are *SLC2A14*, *TRPM7*, and *UGP2*.

*SLC2A14* encodes the protein GLUT14, a member of the glucose transporter (GLUT) family, which is involved in the transport of deoxyglucose and dehydroascorbic acid. This gene is expressed in various tissues, including brain tissue and blood (Amir Shaghaghi et al. 2017; Albaik et al. 2024; Wang et al. 2012). Changes in the expression levels of *SLC2A14* have excellent diagnostic potential for determining the presence of ischemic stroke, as recent studies have identified *SLC2A14* as a gene involved in the iron-dependent form of regulated cell death associated with ischemic stroke (Zhang et al. 2022). An increase in the expression level of *SLC2A14* is observed in our results for the Oximetric group. This suggests that in patients from the Oximetric group, a cascade of iron-dependent regulated cell death associated with ischemic stroke is triggered. A possible connection is indicated with transient, milder symptoms of ischemic stroke, which are common in the Oximetric group after CEA. However, a more moderate increase in *SLC2A14* expression is also observed in the Asymptomatic group, where no symptomatic manifestations of ischemic stroke occur after CEA. Therefore, our research did not confirm a correlation between the increase in *SLC2A14* expression and symptomatic manifestations of ischemic stroke. The only group where a decrease in *SLC2A14* expression is observed after CEA is the Symptomatic group. We hypothesize that this decrease is a consequence of a previous ischemic stroke, leading to the activation of a protective ischemic tolerance cascade caused by natural preconditioning.

*TRPM7* is a calcium-permeable ion channel and also an enzyme that plays a key role in several biological processes, including axon development and the regulation of cells in response to hypoxia and ischemia (Turlova et al. 2023; Sun et al. 2009). *TRPM7* provides a link between the metabolic state of cells and intracellular calcium homeostasis in neurons due to its sensitivity to fluctuations in intracellular Mg-ATP levels (Demeuse et al. 2006). This protein plays a crucial role in ischemic and hypoxic neuronal cell death (Inoue et al. 2020; Sun et al. 2020). Potential mediators of cell death following *TRPM7* activation are considered to be calcium/calmodulin-dependent protein kinase II (CaMKII) and the phosphatase calcineurin. In vivo experiments in mice have shown a significant reduction in brain tissue damage and improvements in both short-term and long-term functions after hypoxic-ischemic brain injury following the administration of the specific *TRPM7* blocker, waixenicin A (Turlova et al. 2021). Our results indicate a decrease in the expression levels of *TRPM7* in both the oximetric and symptomatic patient groups. The reduced expression of this gene is associated with a neuroprotective effect on brain tissue, which we can likely attribute to a previously experienced ischemic stroke in the symptomatic group, followed by tolerance induced by natural preconditioning. In the oximetric group, the decrease is likely associated with short-term mild hypoxia caused by a drop in oximetry levels of more than 20% during CEA, which triggers the body’s defense mechanism to induce ischemic tolerance. In the asymptomatic group, which has not previously experienced any ischemic event, CEA did not cause any significant change in the expression levels of the *TRPM7* gene compared to negative control samples. Therefore, we believe that if a patient has not previously experienced ischemic stroke, the CEA procedure itself, without complications such as a drop in oximetry by more than 20%, is not a sufficient stimulus to trigger ischemic tolerance in the form of a decrease in TRPM7 expression levels.

The enzyme uridine diphosphate-glucose pyrophosphorylase (*UGP2*) is involved in cell proliferation and survival. Microarray analyses have revealed that in atherosclerotic plaques in humans (Zhang et al. 2025), the expression level of this gene is reduced. An increase in UGP2 expression in endothelial cells leads to a decrease in reactive oxygen species (ROS) levels, cleaved caspase-3 expression, and apoptosis rates. These findings suggest that *UGP2* may have a protective effect on endothelial cells and could be an important regulator of cell viability and apoptosis (Zhang et al. 2025). *UGP2* is also the only enzyme that catalyzes the conversion of glucose 1-phosphate to UDP-glucose, which is an important molecule in anabolic pathways. This reaction is a key step in the synthesis of glucose 6-phosphate and the subsequent formation of glycogen, glycolysis, and other metabolic processes that are essential for cellular energy and function (Knop and Hansen 1970). Inhibition of *UGP2* thus leads to a significant reduction in perfusion recovery and vessel density after hypoxia (Ganta et al. 2023). Our results indicate a significant increase in the expression level of *UGP2* in the oximetric group and, to a lesser extent, in the asymptomatic group of patients. We hypothesize that the significant increase in the oximetric group is triggered by a drop in oxygen saturation of more than 20% during CEA, which likely stimulated a reduction in ROS levels and apoptosis, as well as perfusion recovery after hypoxia influenced by the presence of *UGP2* and the neuroprotective cascades it mediates. This neuroprotective effect was observed at a lower intensity in the asymptomatic group as well. Since this group has never experienced ischemic stroke, we assume that the stimulus induced by CEA alone, without complications of oxygen saturation drop above 20%, was sufficient to trigger the neuroprotective response leading to ischemic tolerance. In the symptomatic group of patients, the increase in *UGP2* expression was only observed at a very mild, statistically insignificant level. This may be due to the aftermath of a previous ischemic stroke in this group, which likely caused an increased level of ischemic tolerance induced by natural preconditioning. We assume that if the saturation drop during CEA does not exceed 20%, it is not a sufficient stimulus to significantly activate neuroprotective cascades in this group of patients.

The genes induced in the results of the microarray analysis specific to the Asymptomatic group in our study are *PLLP* and *ND4L*.

Plasmalipin (*PLLP*) is a membrane protein found in the myelin sheath as its main component. *PLLP* also plays an important role in the development and optimal function of the nervous system (Yaffe et al. 2015). It is also involved in intracellular transport, lipid raft formation, and Notch signaling (Shulgin et al. 2021). *PLLP* expression is characteristic of cells that form the nervous system, gastrointestinal tract, and kidneys, with the highest levels of *PLLP* observed in epithelial, CNS, and PNS cells. *PLLP* plays a critical role in the biogenesis of the myelin membrane and myelination, and is involved in the development and maintenance of the nervous system throughout life. Mechanisms associated with nerve regeneration after injury may also include *PLLP*. A direct correlation has been observed between the intensity of remyelination and the expression of *PLLP* mRNA and protein (Shulgin et al. 2021; Azzaz et al. 2023). Our results indicate a significant increase in the expression level of *PLLP* in the asymptomatic group and, to a lesser extent, in the oximetric group of patients. We assume that the marked increase in the asymptomatic group is triggered by the surgical procedure CEA, which, as a stress stimulus, activated the upregulation of genes involved in remyelination processes and brain tissue regeneration. Since the increased expression of *PLLP* in the oximetric group did not exceed the levels observed in the asymptomatic group, we assume that this acute activation of a standby mode is not related to the degree of oxygen saturation drop of more than 20%, but rather to the CEA procedure itself. In contrast, in the symptomatic group, we observed a decrease in *PLLP* expression, which may be associated with the phenomenon of natural preconditioning, which symptomatic patients experienced after overcoming ischemic stroke, which induced ischemic tolerance. In these patients, some pathways are expressed more than in healthy individuals, while others are suppressed. Since we observe a decrease in neuroprotective remyelination cascades managed by *PLLP* activation, we assume that this cascade was suppressed during natural preconditioning.

*ND4L* is a gene that encodes a mitochondrial protein (NADH dehydrogenase (ubiquinone) 1 alpha subcomplex 4 L), which is a subunit of mitochondrial complex I. Complex I is involved in the electron transport chain, which plays a key role in cellular energy production. *ND4L*, as a subunit of complex I, plays an important role in electron transfer within this complex. Its proper function is essential for the efficient operation of the electron transport chain and the subsequent production of ATP (Gowda et al. 2023). The connection between *ND4L* and ischemic stroke occurs through epigenetic mechanisms such as DNA methylation. Overall DNA methylation levels are generally increased after ischemia, associated with heightened activity of DNA methyltransferases (DNMTs) (Zeng et al. 2020). Increased DNA methylation following ischemic injury leads to transcriptional repression of many genes, which exacerbates brain damage. In contrast, DNA demethylation after ischemic injury is associated with recovery processes following a stroke, such as neurogenesis, angiogenesis, gliogenesis, axon growth, and synaptic plasticity. These processes are characterized by an increase in the mRNA levels of genes related to mitochondrial function, including subunits of complex I, such as *ND4L* (Choi et al. 2022). Our results indicate a significant increase in the expression levels of *ND4L* in the oximetric group and, to a lesser extent, in the asymptomatic group of patients. We hypothesize that the significant increase in the oximetric group is triggered by a drop in saturation of more than 20% during CEA, which likely induced a reduction in DNA methylation followed by an increase in mitochondrial function. This neuroprotective effect was also observed in a milder form in the asymptomatic group. We hypothesize that this group, which has never previously experienced ischemic stroke, does not require such a strong stimulus to induce a similar effect, and that even the CEA procedure, without major complications such as a significant drop in saturation, was sufficient to reduce DNA methylation and subsequently increase mitochondrial function. In contrast, the symptomatic group likely exhibited a mild decrease in *ND4L* expression, probably as a result of previously experienced ischemic stroke, suggesting a slight increase in DNA methylation, which is proportional to the reduction in mitochondrial function.

The genes induced in the results of the microarray analysis in our study, specific to the Oximetric group, are *HMSD*, *SESN3*, and *DPY19L4*.

Serpin-domain containing protein is a protein encoded by the *HMSD* gene. It is believed that this protein, containing a minor serpin domain of histocompatibility, functions as an inhibitor of serine proteases (known as serpins). Serpins are known for their ability to inhibit serine proteases, a class of enzymes involved in various physiological processes, including blood clotting, inflammation, and immunity. By inhibiting serine proteases, serpins help regulate these processes and maintain homeostasis in the body. The specific function of the protein encoded by *HMSD* containing the histocompatibility serpin domain likely involves the modulation of serine protease activity in myeloid cells, a type of immune cell involved in innate immunity, as *HMSD* is predominantly expressed in myeloid cells. By regulating the activity of serine proteases in myeloid cells, this protein may influence immune responses, inflammation, and potentially other cellular processes. Compounds that modulate the activity of serine proteases generally exhibit neuroprotective activity (Lebeurrier et al. 2004; Vivien and Buisson 2000). Our results indicate a significant increase in the expression level of *HMSD* in the oximetric group and, to a lesser extent, in the asymptomatic group of patients. We hypothesize that the pronounced increase in the oximetric group is triggered by a drop in oxygen saturation of more than 20% during CEA, which likely caused an increase in the inhibition of serine proteases, followed by the regulation of inflammatory and immune neuroprotective responses. This neuroprotective effect was observed at a lower intensity in the asymptomatic group of patients as well. Since this group has never experienced ischemic stroke, we assume that the stimulus induced by the CEA procedure alone, without complications and with a decrease in oxygen saturation of more than 20%, was sufficient to trigger the body’s neuroprotective response, leading to ischemic tolerance. In the symptomatic group of patients, we did not observe an increase in *HMSD* expression, and its expression level remained the same as in individuals from the negative control group. This may be due to the past ischemic stroke experienced by this group, which likely caused an increased level of ischemic tolerance induced by natural preconditioning. We assume that if oxygen saturation does not drop by more than 20% during CEA, it is not a sufficient stimulus to activate neuroprotective cascades modulating serine protease activity in a significantly sufficient manner in this group of patients.

*SESN3*, or Sestrin 3, is a gene that codes for a protein playing a key role in stress responses and maintaining cellular homeostasis. This protein is a member of the stress-inducible family of sestrin proteins, which reduces intracellular reactive oxygen species (ROS) levels, thereby inducing resistance to oxidative stress (Budanov et al. 2004; Zamkova et al. 2013). Due to its antioxidant biological activity and ability to promote autophagy, the protective effect mediated by sestrins has great potential in the treatment of many neurodegenerative diseases and neurological disorders. *SESN3* is highly expressed in brain tissue, and its expression is often associated with conditions such as seizures, neuropathic pain, or ischemic stroke (IS). These close associations with neurological conditions suggest an important role for *SESN3* in protecting the nervous system and its responses to various stressors and damage (Chen et al. 2022). Our results indicate a significant increase in the expression levels of *SESN3* in the oximetric group, and to a lesser extent in both the asymptomatic and symptomatic groups of patients. We hypothesize that the significant increase observed in the oximetric group is induced by a drop in oxygen saturation of more than 20% during CEA, which likely acts as a strong stressor, inducing an increase in *SESN3* production as part of the sestrin family. This, in turn, reduces intracellular ROS levels and triggers a neuroprotective effect through a cascade of responses that induce resistance to oxidative stress. This neuroprotective effect was observed with significant intensity, though at a lower level, also in the asymptomatic group. Since this group has never experienced ischemic stroke, we assume that the CEA procedure alone, even without complications such as a drop in oxygen saturation of more than 20%, was a sufficient stressor to trigger the stress-induced neuroprotective response, enhancing resistance to oxidative stress through *SESN3*-regulated reduction of intracellular ROS levels. In the symptomatic group of patients, an increase in *SESN3* expression was also observed as a result of the CEA procedure, although this increase was not statistically significant. This may suggest that natural preconditioning, which we hypothesize occurred in the symptomatic group, led to a situation where the stressor induced by CEA, without a drop in oxygen saturation of more than 20%, was not sufficient to induce a stress-induced neuroprotective response at a statistically significant level. However, the mild increase without significant statistical significance suggests that a mild form of activation of this *SESN3*-regulated antioxidant protection against ROS likely occurs.

*DPY19L4* is a gene that encodes a putative C-mannosyltransferase. This enzyme mediates the C-mannosylation of tryptophan residues on target proteins. It is involved in post-translational modifications and is thought to enable mannosyltransferase activity. It has been shown that protein glycosylation, including C-mannosylation, affects the outcome of a cerebrovascular accident by influencing inflammatory response, excitotoxicity, neuronal apoptosis, and disruption of the blood-brain barrier (Shcherbakova et al. 2017). Glycosylation can modify the function, stability, and interactions of proteins, which can impact various cellular processes. In the context of a cerebrovascular accident, altered protein glycosylation may contribute to the inflammatory response, excitotoxicity, and neuronal apoptosis, which can worsen damage after a stroke. Furthermore, *DPY19L4* may be involved in disrupting the blood-brain barrier, leading to increased brain oedema and a worse outcome in stroke (Li et al. 2023). Our results indicate a significant increase in the expression levels of *DPY19L4* in the oximetric group and, to a lesser extent, in the asymptomatic group of patients. We assume that the significant increase in the oximetric group was induced by a drop in oximetry by more than 20% during CEA, which likely led to an increase in mannosyltransferase activity, contributing to the propagation of neurodegenerative cascades affecting inflammation, excitotoxicity, disruption of the blood-brain barrier, and neuronal apoptosis. This neurodegenerative effect was also observed in a milder form in the asymptomatic group. We assume that the lower intensity of the expression increase, indicating a milder course of neurodegenerative processes induced by the increase in mannosyltransferase activity, is related to the degree of stress the patients underwent during CEA. Since the oximetry level in the asymptomatic group did not drop by more than 20%, as in the oximetric group, this form of CEA represents a lower burden on the body, corresponding to a milder intensity of neurodegenerative cascades. In the symptomatic group of patients, however, we observed a significant decrease in the expression of *DPY19L4*. We hypothesize that the ischemic stroke these patients had experienced functions as a form of natural preconditioning, which induced an ischemic-tolerant phenotype. Therefore, we believe that the stressor in the form of CEA, which triggered neurodegenerative processes in patients without a tolerant phenotype, in the case of patients with a tolerant phenotype, initiates a decrease in mannosyltransferase activity, thus dampening neurodegenerative cascades affecting inflammation, excitotoxicity, disruption of the blood-brain barrier, and neuronal apoptosis. This results in the induction and likely the deepening of the already existing neuroprotective effect of ischemic tolerance.

The genes inhibited in the microarray analysis results common to all tested groups are *UBE3A* and *PCDH9*.

The *UBE3A* gene encodes a protein called ubiquitin-protein ligase E3A, also known as E6-associated protein (E6-AP), which plays a key role in the targeted degradation of proteins in cells. This gene is essential for the normal development and function of the nervous system, regulating the synthesis and degradation of proteins to maintain proteostasis (Chaudhary et al. 2023). *UBE3A* has been identified as a regulator of the transcription factors IRF1 and IRF4 (interferon regulatory factors 1 and 4), which are involved in immune reactivity and neuronal survival in brain tissue following ischemic stroke. Several studies have shown that IRF1 acts as a coregulator of p53 in the apoptosis pathway (Furumai et al. 2019). Our results indicate a significant decrease in *UBE3A* expression in the symptomatic group, and a less significant decrease in the asymptomatic group. This significant reduction in expression in the symptomatic group is likely related to the presumed presence of an ischemic tolerant phenotype, which we expect in this group based on their history of ischemic stroke, acting as a form of natural preconditioning. We believe that the intensity of the stress stimulus induced by the CEA procedure was sufficient for conditioned patients to trigger a neuroprotective response through the reduction of *UBE3A* levels, which is involved in the regulation of immune response cascades, neuronal survival, and apoptosis. A similar neuroprotective effect, though not statistically significant, was observed in the asymptomatic group. For this group as well, we hypothesize that the intensity of the stress stimulus induced by CEA was sufficient to induce a neuroprotective response by lowering *UBE3A* levels and subsequently activating the ischemic tolerant phenotype. In the oximetric group, which experienced a drop in oxygen saturation of more than 20% during CEA, we observed a slight increase in *UBE3A* expression. We assume that a drop in oxygen saturation by more than 20% represents too strong a stressor for the patient to respond with the induction of ischemic tolerance through *UBE3A*-regulated cascades. Instead, we see mild neurodegeneration in the brain tissue, which, although statistically significant, is likely not pronounced.

Protocadherin 9 is a member of the protocadherin family and the cadherin superfamily, which are transmembrane proteins containing extracellular domains (Xiao et al. 2018). Cadherin domains mediate cell adhesion in neural tissues in the presence of calcium. The protein encoded by *PCDH9* may therefore be involved in signaling at neuronal synaptic connections (Watanabe et al. 2015). In addition, it was found that *PCDH9* is downregulated 21 hours after treatment of coronary arteries with minimally oxidized LDL (moxLDL), which is a significant risk factor for the development of an atherosclerotic plaque and subsequent increased risk of ischemic stroke, as moxLDL activates immune responses that sustain chronic inflammatory reactions characteristic of atherosclerosis (Karagiannis et al. 2013). Our results point to the downregulation of *PCDH9* across all groups. The most statistically significant decrease in expression was observed in the asymptomatic group. This group of patients, without prior ischemic conditioning, appears to be the most vulnerable to the stress stimulus applied in the form of CEA. This is likely because the stressor activates inflammatory and immune responses in which *PCDH9* participates, similar to the formation of an atherosclerotic plaque, leading to neurodegenerative damage to brain tissue. In the symptomatic group, we observed a very slight decrease in *PCDH9* expression, and these measured values do not differ significantly from the values of the negative control group. The previously experienced ischemic stroke in the symptomatic group of patients can be considered a form of natural preconditioning. Thus, we assume the presence of a tolerant phenotype in this group. This tolerant phenotype helps mitigate or completely block the onset of neurodegenerative cascades related to inflammatory and immune responses in which *PCDH9* is involved. In the oximetric group, we observed a mild but significant downregulation of *PCDH9* due to the CEA procedure with a drop in oximetry of more than 20%. Since the oximetric group consists of both symptomatic and asymptomatic patients, we believe that the resulting *PCDH9* expression reflecting the presence or absence of neurodegenerative cascades is an average value of both patient groups (with and without natural preconditioning), which is supported by the large variability in statistical significance. We hypothesize that for the activation of these specific neurodegenerative pathways associated with *PCDH9* downregulation, the degree of oximetry decline is not as significant a factor as whether ischemic tolerance through preconditioning is present in the patients or not.

In conclusion, the findings of this study suggest that monitoring changes in gene expression in peripheral blood may be useful in identifying an increased ability to induce ischemic tolerance. We have also demonstrated that CEA may initiate protective processes to some extent after ischemic stroke. Based on this, we can consider it a form of ischemic tolerance activation. These insights may contribute to the development of new therapeutic strategies aimed at improving the prognosis of patients with ischemic stroke, for example, through a personalized approach to treatment and the prevention of secondary ischemic events.

## Data Availability

The datasets generated during and/or analyzed during the current study are not publicly available but are available from the corresponding author on reasonable request.

## Declarations

### Submission Declaration and Verification

The work has not been published previously, it is not under consideration for publication elsewhere, its publication is approved by all authors and tacitly or explicitly by the responsible authorities where the work was carried out, and, if accepted, it will not be published elsewhere in the same form, in English or in any other language, including electronically without the written consent of the copyright-holder.
